# Risks and benefits of giving early Aspirin within 6 hours of CABG: A retrospective analysis

**DOI:** 10.12669/pjms.331.11563

**Published:** 2017

**Authors:** Muhammad Yasir Khan, Adnan Zafar Khan, Anjum Jalal, Haider Zaman

**Affiliations:** 1 Dr. Muhammad Yasir Khan, MCPS, FCPS, FCPS, MRCS. Department of Cardiac Surgery, Chaudhary Pervaiz Elahi Institute of Cardiology, Multan, Pakistan; 2 Dr. Adnan Zafar Khan, MBBS, MSc. Health economics. Department of Health Punjab, Lahore, Pakistan; 3 Prof. Anjum Jalal, FRCS, FCPS (CS), FRCS Cth. Department of Cardiac Surgery, Institute of Cardiology Faisalabad, Pakistan; 4 Prof. Haider Zaman, FCPS, FRCS Cth. Department of Cardiac Surgery, Chaudhary Pervaiz Elahi Institute of Cardiology, Multan, Pakistan

**Keywords:** Aspirin, Antiplatelet drugs, Coronary artery bypass surgery

## Abstract

**Background & Objective:**

Antiplatelet drugs are frequently used after coronary artery bypass graft (CABG) surgery to prevent venous graft occlusion. The fear of bleeding complications prevents them to be given early post operatively, which is the time when antiplatelets use confers maximum benefit. Our objective was to determine the effect and influence of early aspirin therapy on fatal and nonfatal bleeding complications and blood requirements after coronary bypass surgery (CABG).

**Methods:**

The patients who only underwent coronary artery bypass surgery for the first time in the past three years and did not have any bleeding diathesis were retrospectively analyzed from the cardiac surgery database of CPEIC Multan. The patients either received aspirin within six hours of CABG or had it given after 12 hours. The patients were analyzed for mean blood loss and number of blood units transfused. SPSS was used for statistical analysis. P value < 0.05 was considered significant.

**Results:**

Total 281 patients received aspirin within six hours while 326 patients did not. Mean blood loss in early aspirin group was 727ml as compared to 767ml in the other group (p value 0.74). The median number of blood units transfused was 2 (p value 0.98). Our results did not show any statistical difference in both the groups.

**Conclusion:**

Aspirin can safely be given early after CABG without the fear of bleeding complications thus conferring the advantage of increased graft patency.

## INTRODUCTION

The success of coronary artery bypass grafting depends largely upon the patency of the vascular grafts post operatively. It includes both the arterial and the venous grafts. Though the surgical technique in addition to the graft quality and the coagulation state of the patient plays a pivotal role in preventing graft occlusion, the role of antiplatelet drugs cannot be ignored. Because platelet activation constitutes a pivotal mechanism for graft occlusion post operatively, the role of anti-platelet drugs becomes substantial as they will reduce the formation of thrombus, prevent graft occlusion, and protect graft patency. Aspirin, owing to its anti-platelet action, is one of the most cost-effective therapies available for the prevention and treatment of platelet-mediated vascular occlusive disorders, which consist of a heterogeneous group of diseases including myocardial infarction (MI), stroke, and vascular graft thrombosis. Some 8-18% of the venous grafts are occluded in the first month post operatively and the cause is graft thrombosis.

The major indication for aspirin after cardiac surgery is to reduce the incidence of vein graft occlusion early after CABG. In a meta-analysis of 20 randomised clinical trials that included more than 5000 patients who underwent CABG, anti-platelet therapy reduced the proportion of patients who suffered graft occlusion by 41% (21.1% vs. 30.3%, P < 0.00001).^1^ It is only due to this fact that timing of giving aspirin is most important. According to the AHA and ESC guidelines^2^,^3^ it should be given within 6 hours post-operatively but concern about bleeding has been paramount and has resulted in the reluctance to give it early post operatively by many surgeons.

To address these issues, we conducted a retrospective study to determine the effect and influence of early aspirin therapy on fatal and nonfatal bleeding complications and blood requirements after coronary bypass surgery (CABG).

## METHODS

This is a retrospective observational study conducted at Ch Pervaiz Elahi Institute of Cardiology Multan, Pakistan. The data of all the patients who had only coronary artery bypass grafting surgery for the first time in the last three years was retrieved from the cardiac surgery database. The patients who had valvular heart surgery, coagulopathies, congenital heart defects and all other patients having additional procedures with CABG were excluded from the study. The aspirin was stopped at least five days pre-operatively in all these patients. Because of the two schools of thoughts in the department, the patients either received 150mg of aspirin within 6 hours of surgery or the following day after 12 hours of coronary artery bypass grafting. A total of 607 patients (n=607) met the inclusion criteria and were analysed by dividing them in two groups. Group 1 patients received aspirin within six hours of surgery while the second group had aspirin after 12 hours of operation. None of these patients received clopidogrel or any other anti-platelet in addition to aspirin post-operatively. Standard technique of cardiopulmonary bypass was used in all the patients. The groups were compared in terms of post operative blood loss and the number of whole blood used. All these patients were followed up till the time of discharge. Categorical variables were analysed using Chi sq. test while quantitative data was compared by Mann Whitney U test. The statistical analysis was done using SPSS.

## RESULTS

Six hundred and seven patients met the inclusion criteria. Three hundred twenty seven patients did not receive aspirin in 6 hours while 280 patients had it given in 6 hours of CABG. Mean age in both the groups was 55 and 54.79 years respectively. About 15.2% of the patients were female while 84.8% of the patients were male. Age and gender distribution are explained in detail in Tables I and II respectively. The patients in both the groups were statistically comparable in terms of age, gender, and bypass time, cross clamp time, ejection fraction, CK MB and number of grafts as is shown in Table-III.

**Table-I:** Age.

Group Statistics					

Age	Aspirin	N	Mean	Std. Deviation	Std. Error Mean
	No	326	55.00	9.665	0.534
	Yes	281	54.79	9.345	0.558

**Table-II:** Gender.

Aspirin * Gender Cross tabulation					

			Gender		Total

			Female	Male	
*Aspirin*	*No*	Count	54	272	326
		% of Total	8.9%	44.8%	53.7%
	*Yes*	Count	38	243	281
		% of Total	6.3%	40.0%	46.3%
	*Total*	Count	92	515	607
		% of Total	15.2%	84.8%	100.0%

**Table-III:** Comparison of patient characteristics pre-op and post-op.

Variables	No Aspirin (after 6 hrs)	Yes Aspirin (in 6 hrs)	P value
Total number n	327	280	
Age mean (years)	55	54.79	0.79
Gender			
Male	272(44.8%)	243 (40%)	0.30
Female	54 (8.9%)	38 (6.3%)	
Ejection fraction (mean %)	51.35	50.78	0.41
Bypass time (mean min)	109.38	108.25	0.50
Cross Clamp time (mean min)	64.10	64.31	0.93
No of grafts (mean)	3	3	0.23
Max CKMB post op (mean)	51.92	51.19	0.87

**Fig. 1:**
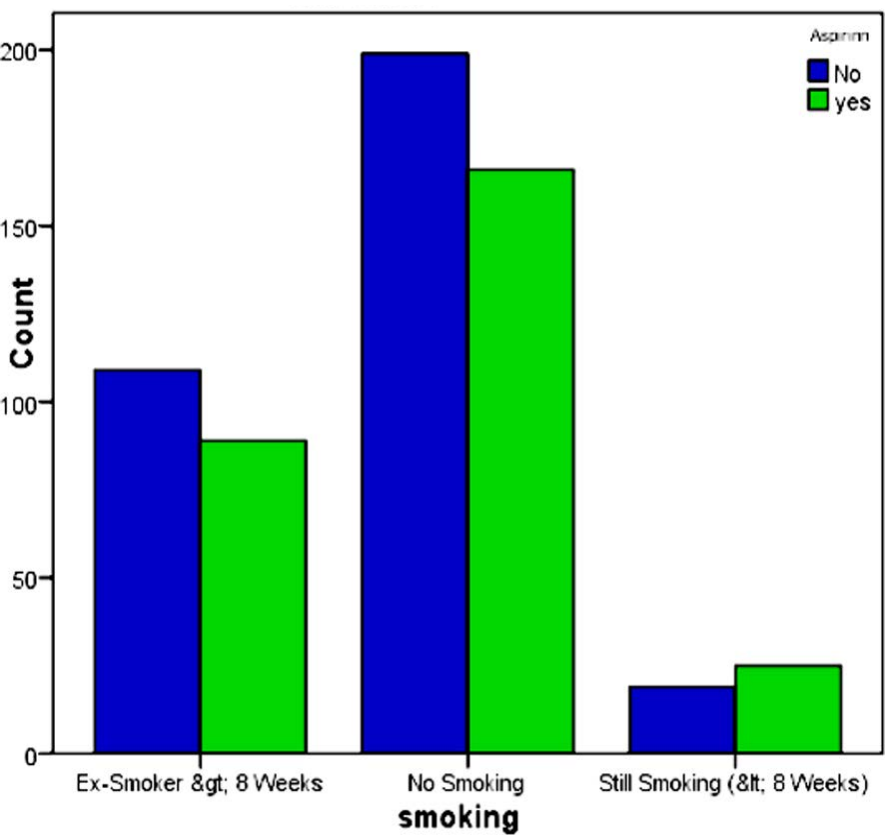
Frequency of smokers, ex-smokers and non smokers in both the groups.

**Fig. 2:**
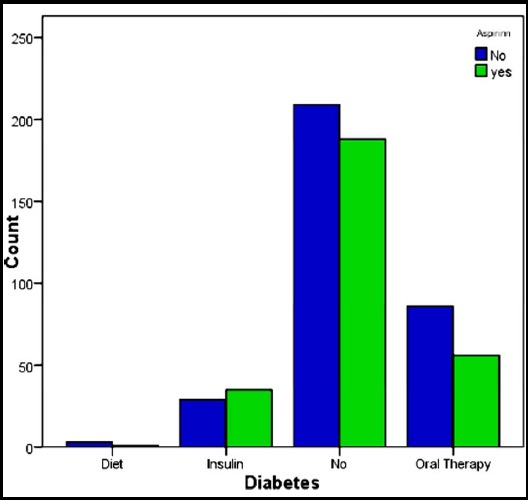
Frequency of diabetics and non diabetics in both the groups.

Mean blood loss in group 1 was 767ml while it was 727ml in the patients who received early aspirin within 6 hours of CABG. When both the groups were compared for blood loss and number of units transfused, no statistically significant difference observed with a p-value of 0.74. The median number of blood units transfused in both the groups was two with a p-value of 0.98 again showing no statistical significance. This is shown in Table-IV (a). Table-IV (b) shows the test statistics (p values) of different variables. The frequency of smoking and diabetes among both the groups is clearly depicted in Fig. 1 and 2 respectively. Our results clearly depicts that giving aspirin early (within 6 hours) after CABG pose no increased risk of bleeding or the number of blood units transfused.

**Table-IV a:** Comparison of outcome variables.

Variables	No Aspirin (after 6 hrs)	Yes Aspirin (in 6 hrs)	P value
Chest drainage (mean)	767ml	727ml	0.74
No of Blood units used (mean)	2.3	2.4	0.98

**Table-IV b:** Test Statistics of different variables.

	Distal	BMI	Cpb time	Cx time	MaxCKMB	RBC2	Chest Drainage
Mann-Whitney U	43389.000	45657.000	43510.000	44736.500	42605.000	45750.000	45084.500
Wilcoxon W	82729.000	99285.000	82570.000	96739.500	93326.000	85090.000	98712.500
Z	-1.181	-0.057	-0.664	-0.086	-0.158	-0.015	-0.323
Asymp. Sig. (2-tailed)	0.238	0.954	0.507	0.931	0.874	0.988	0.747
Exact Sig. (2-tailed)	0.238					0.988	
Exact Sig. (1-tailed)	0.119					0.494	
Point Probability	0.000					0.000	

a. Grouping Variable: Aspirin

b. Some or all exact significances cannot be computed because there is insufficient memory.

## DISCUSSION

The results of our study clearly shows that there was no statistically significant difference in blood loss and the number of blood units transfused when aspirin is given either within or after 6 hours of CABG. We know that early aspirin therapy after CABG increases the graft patency. According to the VA trial all aspirin-containing therapeutic regimens improved (p less than 0.05) graft patency compared with placebo (85.2%).^4^

Despite this the surgeons are still reluctant to prescribe early aspirin after CABG because of two reasons. Firstly the platelet number and function is markedly reduced post operatively due to sequestration and hypothermia^5^ and mechanical filtration. Secondly because of reduced number and function the addition of aspirin would cause excessive bleeding.

The paramount importance of the timing of administering aspirin is evident from several studies. Gavaghan et al., in a double-blind placebo-controlled randomised study showed aspirin when given within one hour of CABG reduced the incidence of early and late graft occlusion. This study also demonstrated that early aspirin is not associated with post op bleeding or increased transfusion.^6^ Frames et al. in a meta-analysis of 17 randomised studies comparing 4504 patients showed that early aspirin does not cause significant post operative bleeding but enhances early vein graft patency.^7^ Our results are also consistent with these rather mean blood loss was slightly lower in the group having early aspirin; 727ml vs.767ml in the other group.

The earlier it’s given post CABG the more the benefit is conferred in terms of graft patency. In a prospective randomised double-blind trial by Sharma et al. no benefit of early aspirin on vein graft patency was demonstrated when given after 48 hours.^8^ Likewise in a systematic review of a wide range of issues in CABG, Eagle et al. found out that aspirin significantly reduces graft occlusion if given at 1,7 or 24 hours but not at 48 hours.^9^ It is well known that intimal hyperplasia and vein graft atherosclerosis are not influenced by aspirin therapy, the beneficial effects of aspirin are not therefore seen after the first year of CABG.

On the contrary preoperative ingestion of aspirin is associated with increased post operative bleeding complications. In a multi centre cohort study Kremke et al. concluded that preoperative anti platelet is associated with increased bleeding and greater transfusion requirements after CABG. Clopidogrel was found to be an independent risk factor for severe post operative bleeding.^10^ Similarly Goldman et al in a multi centred randomised trial showed that preoperative aspirin is associated with more bleeding, transfusion requirements. They also found out that early post operative aspirin increases graft patency and is not associated with post operative bleeding unlike preoperative aspirin.^11^

Early aspirin use not only increases graft patency but it has also been demonstrated to reduce the mortality and major adverse effects after CABG. In a prospective study by Mangano et al., the relationship between early aspirin use and fatal and non fatal outcomes was studied. Among patients who received early aspirin within 48 hours of revascularization, mortality was 1.3% as compared to 4.0% in the placebo group. Aspirin therapy was associated with a 48% reduction in the incidence of myocardial infarction, a 50% reduction in the incidence of stroke, a 74% reduction in the incidence of renal failure and a 62% reduction in the incidence of bowel infarction.^12^

Despite this compelling evidence of early aspirin administration post CABG, there is a reluctance to follow the guidelines. Gukop et al., studied the reasons of deviation from these guidelines in 200 consecutive patients. They found out that post operative bleeding was the leading cause of non administration of early aspirin at 6 hours. This study did not demonstrate any significant difference in the in blood loss and transfusion requirements between early aspirin and non administered group.^13^ Again supporting the results of our study.

## CONCLUSION

We therefore recommend that aspirin should be given within 6 hours of CABG in accordance with the AHA and European guidelines.

***Grant Support & Financial Disclosures:*** None.
